# Derivation and Validation of a Phenoconversion‐Related Pattern in Idiopathic Rapid Eye Movement Behavior Disorder

**DOI:** 10.1002/mds.29236

**Published:** 2022-10-03

**Authors:** Pietro Mattioli, Beatrice Orso, Claudio Liguori, Francesco Famà, Laura Giorgetti, Andrea Donniaquio, Federico Massa, Andrea Giberti, David Vállez García, Sanne K. Meles, Klaus L. Leenders, Fabio Placidi, Matteo Spanetta, Agostino Chiaravalloti, Riccardo Camedda, Orazio Schillaci, Francesca Izzi, Nicola B. Mercuri, Matteo Pardini, Matteo Bauckneht, Silvia Morbelli, Flavio Nobili, Dario Arnaldi

**Affiliations:** ^1^ Department of Neuroscience, Rehabilitation, Ophthalmology, Genetics, Maternal and Child Health (DINOGMI), Clinical Neurology University of Genoa Genoa Italy; ^2^ Department of Radiology and Nuclear Medicine Amsterdam UMC, Location VuMC, Amsterdam Neuroscience Amsterdam The Netherlands; ^3^ Department of Systems Medicine University of Rome “Tor Vergata” Rome Italy; ^4^ Sleep Medicine Center, Neurology Unit University Hospital “Tor Vergata” Rome Italy; ^5^ IRCCS Ospedale Policlinico S. Martino Genoa Italy; ^6^ Department of Neurology University of Groningen, University Medical Center Groningen Groningen The Netherlands; ^7^ Department of Nuclear Medicine and Molecular Imaging, University of Groningen University Medical Center Groningen Groningen The Netherlands; ^8^ Department of Biomedicine and Prevention University of Rome “Tor Vergata” Rome Italy; ^9^ IRCCS Fondazione Santa Lucia Rome Italy; ^10^ Department of Health Science (DISSAL) University of Genoa Genoa Italy; ^11^ IRCCS Neuromed Pozzilli Italy

**Keywords:** rapid eye movement sleep behavior disorder, fluorodeoxyglucose positron emitting tomography, α‐synucleinopathy, phenoconversion, disease‐related pattern

## Abstract

**Background:**

Idiopathic rapid eye movement sleep behavior disorder (iRBD) represents the prodromal stage of α‐synucleinopathies. Reliable biomarkers are needed to predict phenoconversion.

**Objective:**

The aim was to derive and validate a brain glucose metabolism pattern related to phenoconversion in iRBD (iRBDconvRP) using spatial covariance analysis (Scaled Subprofile Model and Principal Component Analysis [SSM‐PCA]).

**Methods:**

Seventy‐six consecutive iRBD patients (70 ± 6 years, 15 women) were enrolled in two centers and prospectively evaluated to assess phenoconversion (30 converters, 73 ± 6 years, 14 Parkinson's disease and 16 dementia with Lewy bodies, follow‐up time: 21 ± 14 months; 46 nonconverters, 69 ± 6 years, follow‐up time: 33 ± 19 months). All patients underwent [^18^F]FDG‐PET (^18^F‐fluorodeoxyglucose positron emitting tomography) to investigate brain glucose metabolism at baseline. SSM‐PCA was applied to obtain the iRBDconvRP; nonconverter patients were considered as the reference group. Survival analysis and Cox regression were applied to explore prediction power.

**Results:**

First, we derived and validated two distinct center‐specific iRBDconvRP that were comparable and significantly able to predict phenoconversion. Then, SSM‐PCA was applied to the whole set, identifying the iRBDconvRP. The iRBDconvRP included positive voxel weights in cerebellum; brainstem; anterior cingulate cortex; lentiform nucleus; and middle, mesial temporal, and postcentral areas. Negative voxel weights were found in posterior cingulate, precuneus, middle frontal gyrus, and parietal areas. Receiver operating characteristic analysis showed an area under the curve of 0.85 (sensitivity: 87%, specificity: 72%), discriminating converters from nonconverters. The iRBDconvRP significantly predicted phenoconversion (hazard ratio: 7.42, 95% confidence interval: 2.6–21.4).

**Conclusions:**

We derived and validated an iRBDconvRP to efficiently discriminate converter from nonconverter iRBD patients. [^18^F]FDG‐PET pattern analysis has potential as a phenoconversion biomarker in iRBD patients. © 2022 The Authors. *Movement Disorders* published by Wiley Periodicals LLC on behalf of International Parkinson and Movement Disorder Society.

Rapid eye movement (REM) sleep behavior disorder (RBD) is a parasomnia characterized by the enactment of dreams and the loss of the physiological muscle atonia during REM sleep.^1^ When not associated with overt neurologic or psychiatric diseases, it is defined as “idiopathic” or “isolated” (iRBD). Nevertheless, iRBD has been described to be the prodromal stage of α‐synucleinopathies.^2^, ^3^, ^4^ Indeed, more than 70% of iRBD patients will eventually phenoconvert to an overt α‐synucleinopathy within 12 years from diagnosis.^2^, ^3^, ^4^ Moreover, the majority of iRBD patients show biological signs of synucleinopathy in the skin nerves and/or in the cerebrospinal fluid.^5^, ^6^ Thus, iRBD patients comprise the ideal target population for testing disease‐modifying therapies once available.^7^ To design such trials, biomarkers able to predict short‐term phenoconversion to the overt stage of alpha‐synucleinopathies are needed. However, the time from diagnosis to phenoconversion is highly variable, with several patients not yet phenoconverted after 10 years,^8^, ^9^ but it has been suggested that disease‐modifying trials should last up to 2 years.^10^ ^18^F‐fluorodeoxyglucose positron emitting tomography ([^18^F]FDG‐PET), when combined with analytical techniques, might be a promising biomarker of phenoconversion, as it has been proved to predict phenoconversion in different neurodegenerative diseases, such as Alzheimer's disease.^11^, ^12^ The scaled subprofile model and principal component analysis (SSM‐PCA) allows to extract the pattern of covariance between voxels, and it has been previously applied to [^18^F]FDG‐PET data to identify disease‐specific brain patterns in several neurological conditions, including iRBD^13^ and Parkinson's disease (PD).^13^, ^14^ Regarding the PD‐related pattern (PDRP),^15^ it has been shown that pattern expression increases with disease progression and decreases with effective therapy.^16^ PDRP is also expressed in iRBD patients at baseline, and it has been proved to increase over time and it is associated with phenoconversion.^14^, ^17^, ^18^ However, PDRP has been identified in PD patients regardless of the presence/absence of RBD. To evaluate disease progression in RBD patients, it might be more useful to use a pattern identified in PD patients with RBD. Recently, an [^18^F]FDG‐PET pattern was derived in a group of de novo PD patients with RBD (dnPDRBD‐RP) and applied in a group of iRBD patients, trying to solve this issue.^15^ The dnPDRBD‐RP significantly predicted phenoconversion to overt alpha‐synucleinopathy, showing better performance than the general PDRP.^15^

This result suggests that [^18^F]FDG‐PET may be a good biomarker for phenoconversion.

A specific phenoconversion‐related pattern, highlighting the brain metabolic differences between converter iRBD patients and nonconverters, may provide a more reliable estimation in identifying iRBD patients at high risk of short‐term phenoconversion, regardless of the final diagnosis.

Using SSM‐PCA analysis, we studied [^18^F]FDG‐PET imaging data of both iRBD patients who subsequently developed an overt α‐synucleinopathy and those who did not (ie, retained the iRBD diagnosis) at follow‐up, to gain insight into the phenoconversion‐related pattern and its ability to predict short‐term phenoconversion. The study was conducted in two centers, each one used to derive the pattern that was then validated in the other center, thus increasing the stability of the results.

## Patients and Methods

### Patients

From two Italian centers, Genoa (GE) and Rome Tor Vergata (RTV), we enrolled 30 iRBD patients (mean age: 73 ± 6, 23 men, follow‐up time: 21 ± 14 months) who subsequently phenoconverted to an overt α‐synucleinopathy (iRBD converters) and 46 iRBD patients (mean age: 69 ± 6, 38 men, follow‐up time: 33 ± 19 months) who did not phenoconvert (iRBD nonconverters). Demographic and clinical data are presented in Tables S1 and S2. Moreover, 44 healthy controls (mean age: 70 ± 8.53, 16 men) and 32 de novo PD patients (mean age: 73.12 ± 5.86, 10 men) with RBD were selected from our data set. Demographic and clinical data are presented in Table S3.

RBD diagnosis was confirmed in both iRBD and de novo PD patients using polysomnography (PSG), according to current criteria.^19^ For de novo PD patients, the diagnosis was performed following current criteria^20^ and confirmed by evidence of dopaminergic deficit on [^123^I]FP‐CIT SPECT and by at least 2 years of follow‐up.

At baseline, all patients underwent the Movement Disorder Society Unified Parkinson's Disease Rating Scale, motor section (MDS‐UPDRS‐III), to investigate the presence of parkinsonism, the Mini Mental State Examination (MMSE), as a measure of global cognitive functioning, as well as a comprehensive neuropsychological assessment, including at least two tests for each of the main cognitive domains (verbal memory, executive functions, attention and working memory, and visuospatial abilities and language)^21^ to evaluate the presence of mild cognitive impairment (MCI).^21^ Patients with dementia and parkinsonism fulfilling criteria for the diagnosis of PD, multiple system atrophy (MSA), or dementia with Lewy bodies (DLB) at baseline (ie, they did not have iRBD) were excluded. Clinical conditions (eg, activities and instrumental activities of daily living assessment, motor and cognitive assessment) were evaluated prospectively every 6 months from baseline. Phenoconversion to overt alpha‐synucleinopathy (ie, PD, DLB, or MSA) was assessed using current criteria.^22^, ^23^, ^24^

All patients provided written consent to the study. The local ethics committee approved the study protocol, and all participants signed an informed consent form in compliance with the Helsinki Declaration of 1975.

### [^18^F]FDG‐PET

All patients underwent [^18^F]FDG‐PET to investigate brain glucose metabolism within 12 months from iRBD diagnosis. Brain [^18^F]FDG‐PET scans were acquired according to the guidelines of the European Association of Nuclear Medicine.^25^

GE and RTV acquisition protocols are described in Supporting Information.

All [^18^F]FDG‐PET images were acquired in static mode and then subjected to affine and nonlinear spatial normalization into Montreal Neurological Institute (MNI) brain space using SPM12 (Wellcome Department of Cognitive Neurology, London, UK). All the default settings of SPM were used, and the specific[^18^F]FDG‐PET brain template was used as reference.^26^

The spatially normalized set of images was then smoothed with a 10‐mm isotropic Gaussian filter to account for individual anatomical variability and to improve the signal‐to‐noise ratio.

### Polysomnographic Recording

Patients underwent overnight PSG. Sleep scoring was performed following current criteria.^19^ PSG derivations were placed according to recommended rules^19^ to evaluate sleep features and respiratory, cardiac, and limb events. Patients were asked to withdraw melatonin, hypnotic medications, and antidepressant drugs for 2 weeks before the recording.

### iRBDconvRP Derivation and Validation among GE and RTV Centers

The phenoconversion‐related pattern of iRBD (iRBDconvRP) patients was derived using an automated algorithm^27^, ^28^ developed by the University Medical Center Groningen (UCMG), the Netherlands, based on the SSM‐PCA method of Spetsieris and Eidelberg^29^ implemented in MatLab (version 2020a, MathWorks, Natick, MA).

We first derived independent patterns for the two centers (GE and RTV) as across‐centers validation test, because having multiple sites often represents an issue.^30^

In brief, SSM‐PCA was first applied to a derivation set (GE patients) of 16 converter and 27 nonconverter patients. The resulting iRBDconvRP was then applied to a validation set (RTV) of 14 converter and 19 nonconverter patients, to confirm its ability to discriminate between converters and nonconverters.

The same process was repeated for the RTV cohort, obtaining the iRBDconvRP from RTV patients and subsequently calculating subject scores in the GE cohort (see Supporting Information for details).

### iRBDconvRP Derivation and Validation on the Whole Patient Group

We pooled the data to identify the iRBDconvRP in the total data set (30 converters and 46 nonconverters). For validation, we performed a leave‐one‐out cross‐validation (LOOCV).^31^, ^32^ Subject scores expressing both the original iRBDconvRP and the LOOCV iRBDconvRP were *z*‐transformed with respect to the nonconverter iRBD patients. Considering the known heterogeneity of iRBD patients,^33^ and to enhance the stability of the results, we performed a bootstrap resampling (2000 repetitions) to extract the most stable regions in the iRBDconvRP. Unthresholded and thresholded voxels (2.5%–97.5% confidence interval [CI]) were overlaid on a T1‐MRI template for visualization.

### DenovoPDRBDRP Derivation

Thirty‐two de novo PD patients with RBD and 44 healthy controls were used to obtain a denovoPDRBDRP, using the same method described in the previous section. denovoPDRBDRP was applied to the iRBD group to obtain the subject scores for each patient. Subject scores were *z*‐transformed in respect to the nonconverter iRBD patients.

### Statistical Analysis

Between‐group differences in clinical characteristics and subjects’ *z* scores were assessed using the unpaired *t* test for continuous variables and the χ2 test for categorical variables.

To determine the sensitivity and specificity of the patterns, a receiver operating curve (ROC) was plotted based on *z*‐transformed subject scores. For the iRBDconvRP, LOOCV *z* scores were used in the analysis. The cutoff that produced optimum sensitivity and specificity, calculated using the Youden index method,^34^ was chosen as the threshold.

Next, Kaplan–Meier survival analysis was performed to estimate the risk of phenoconversion from iRBD to an overt α‐synucleinopathy, using pattern expression values, categorized as below or above the threshold previously computed by the Youden index method. The survival time was set as the interval (expressed in months) between the date of [^18^F]FDG‐PET and the last follow‐up visit in nonconverter patients and between the date of [^18^F]FDG‐PET and the date of phenoconversion in converter patients. The hazard ratio (HR) was calculated with a Cox regression, using age, sex, and site as covariates. For the iRBDconvRP, the presence of MCI and the MDS‐UPDRS‐III score were subsequently added as covariates to explore their possible influence in predicting the phenoconversion.

The aforementioned survival analyses were first performed using the subject scores of GE and RTV groups obtained by applying both the RTV‐iRBDRP and the GE‐iRBDRP on GE and RTV cohorts, respectively. Then, the iRBDconvRP was tested using the subject scores derived from the application of the iRBDconvRP after the LOOCV procedures on the whole group. Subsequently, the denovoPDRBDRP was applied on the whole iRBD group.

Finally, the predictive power of the iRBDconvRP and the denovoPDRBDRP was compared using Cox regression. Moreover, time‐dependent ROCs were calculated, and the area under the curve (AUC) of each time point was compared (one time point every 6 months, until month 48). Partial Pearson's correlation analysis was performed between (1) iRBDconvRP expression and survival time, (2) iRBDconvRP expression and MDS‐UPDRS‐III score, and (3) denovoPDRBDRP and iRBDconvRP expression, using age as a nuisance variable. Binary logistic regression was applied between iRBDconvRP expression and the presence/absence of MCI. Finally, iRBDconvRP expression was compared between iRBD patients with and without MCI.

Statistical threshold was set at 0.05, and *P*‐values were reported to be corrected for multiple comparisons using the Bonferroni approach.

All analyses were performed using MatLab (version 2020a, MathWorks) and Stata software (StataCorp. 2013. Stata Statistical Software: Release 13. College Station, TX: StataCorp LP).

## Results

### Clinical Results

As expected, nonconverter patients were younger, had higher MMSE and lower MDS‐UPDRS‐III scores, and were less frequently affected by MCI, compared with converter patients (Table S1).

RTV patients had a higher MDS‐UPDRS‐III score compared with GE patients (Table S2).

### iRBDconvRP Derivation and Validation

**FIG 1:**
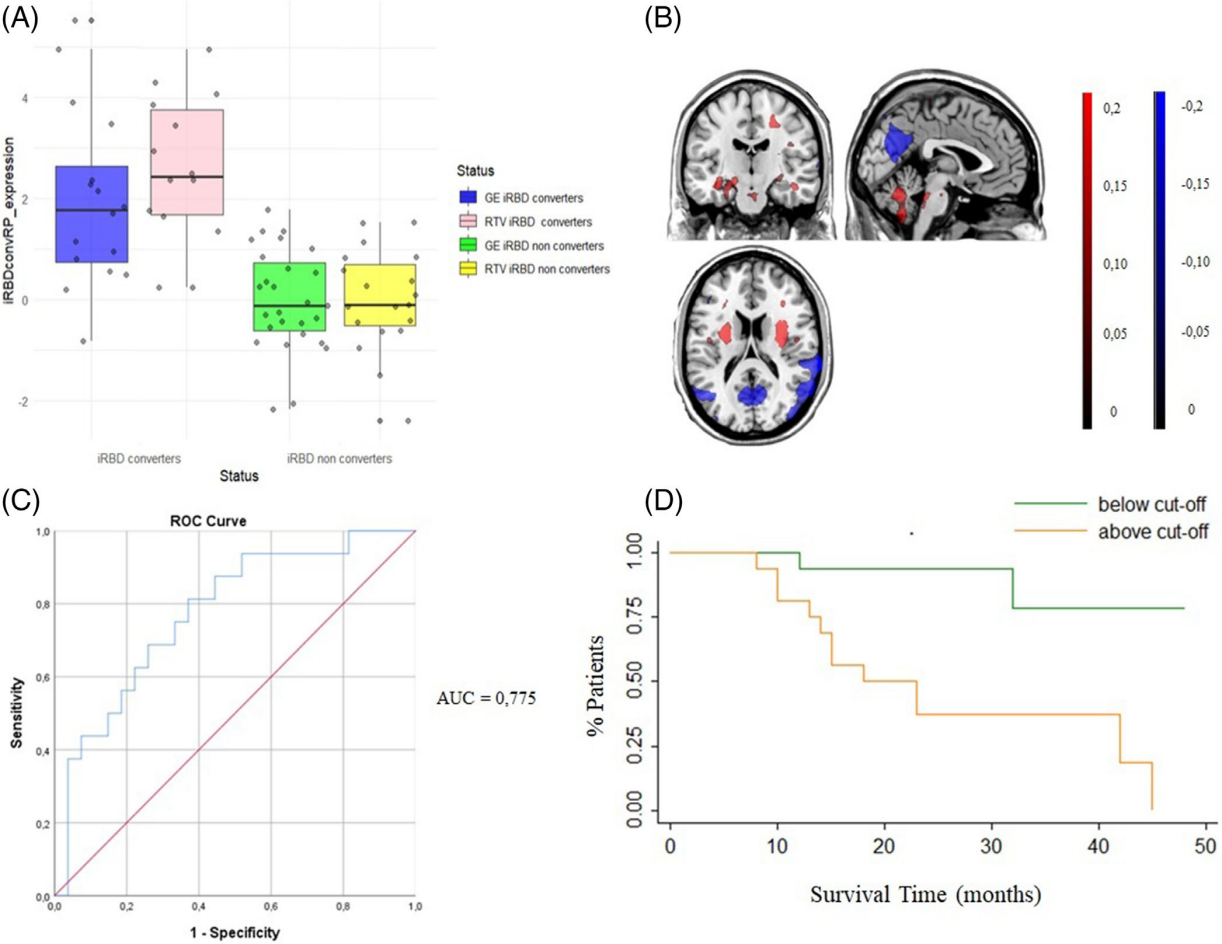
Derivation of the iRBDconvRP in GE group with its validation on RTV group. (**A**) The plot represents the distribution of the GE‐iRBDconvRP expression (*z* score) of the whole voxels in GE converters (blue) and nonconverters (green), and the pattern identified in the GE group applied to the RTV converters (pink) and nonconverters (yellow). (**B**) Results of the SSM‐PCA. Display of stable voxels of the GE‐iRBDconvRP overlaid on a T1‐MRI template in MNI space, determined after bootstrap resampling 95% confidence interval not straddling zero. Red indicates positive voxel weights (relative hypermetabolism), and blue indicates negative voxel weights (relative hypometabolism). (**C**) Results of the ROC analysis performed between converter‐ and nonconverter individual *z* scores (AUC = 0.775). (**D**) Results of the survival analysis of the GE‐iRBDconvRP expression applied to RTV patients. Green line represents GE‐iRBDconvRP expression below the empirical optimal cut point. Orange line represents GE‐iRBDconvRP expression above the empirical optimal cut point. GE, Genoa; iRBDconvRP, phenoconversion‐related pattern of iRBD; ROC, receiver operating curve; RTV, Rome Tor Vergata. [Color figure can be viewed at wileyonlinelibrary.com]

**FIG 2:**
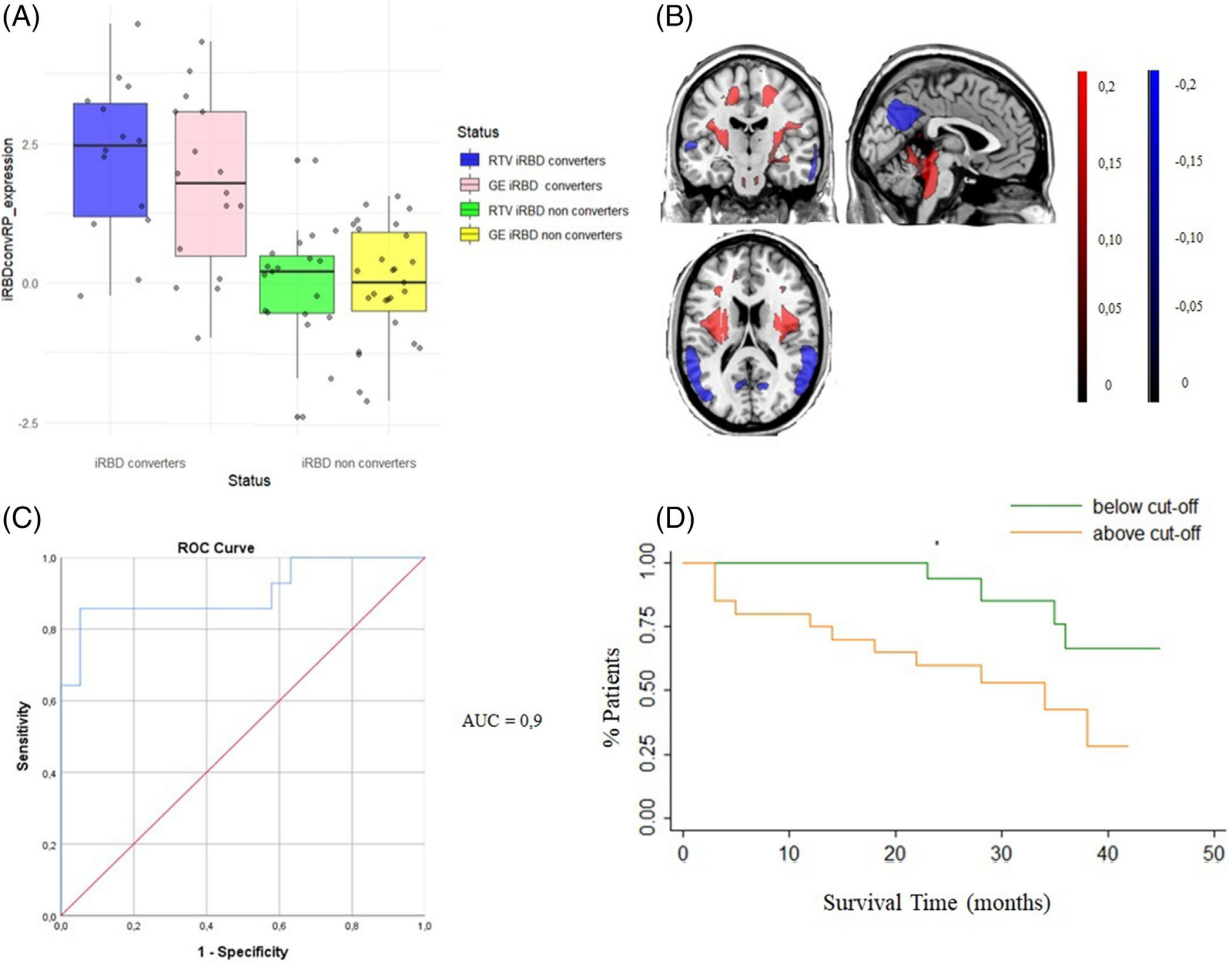
Derivation of the iRBDconvRP in RTV group with its validation on GE group. (**A**) The plot represents the distribution of the RTV‐iRBDconvRP expression (*z* score) of the whole voxels in RTV converters (blue) and nonconverters (green), and the pattern identified in the RTV group applied to the GE converters (pink) and nonconverters (yellow). (**B**) Results of the SSM‐PCA. Display of stable voxels of the RTV‐iRBDconvRP overlaid on a T1‐MRI template in MNI space, determined after bootstrap resampling 95% confidence interval not straddling zero. Red indicates positive voxel weights (relative hypermetabolism), and blue indicates negative voxel weights (relative hypometabolism). (**C**) Results of the ROC analysis performed between converter‐ and nonconverter individual *z* scores (AUC = 0.9). (**D**) Results of the survival analysis of the RTV‐iRBDconvRP expression applied to GE patients. Green line represents RTV‐iRBDconvRP expression below the empirical optimal cut point. Orange line represents RTV‐iRBDconvRP expression above the empirical optimal cut point. AUC, area under the curve; GE, Genoa; iRBDconvRP, phenoconversion‐related pattern of iRBD; MNI, Montreal Neurological Institute; ROC, receiver operating curve; RTV, Rome Tor Vergata. [Color figure can be viewed at wileyonlinelibrary.com]

GE‐iRBDconvRP significantly predicts phenoconversion in RTV patients (HR = 9.29, *P* = 0.004; Fig. 1), whereas RTV‐iRBDconvRP showed a lower, but still‐significant prediction power in GE patients (HR = 3.67, *P* = 0.033; Fig. 2).

**FIG 3:**
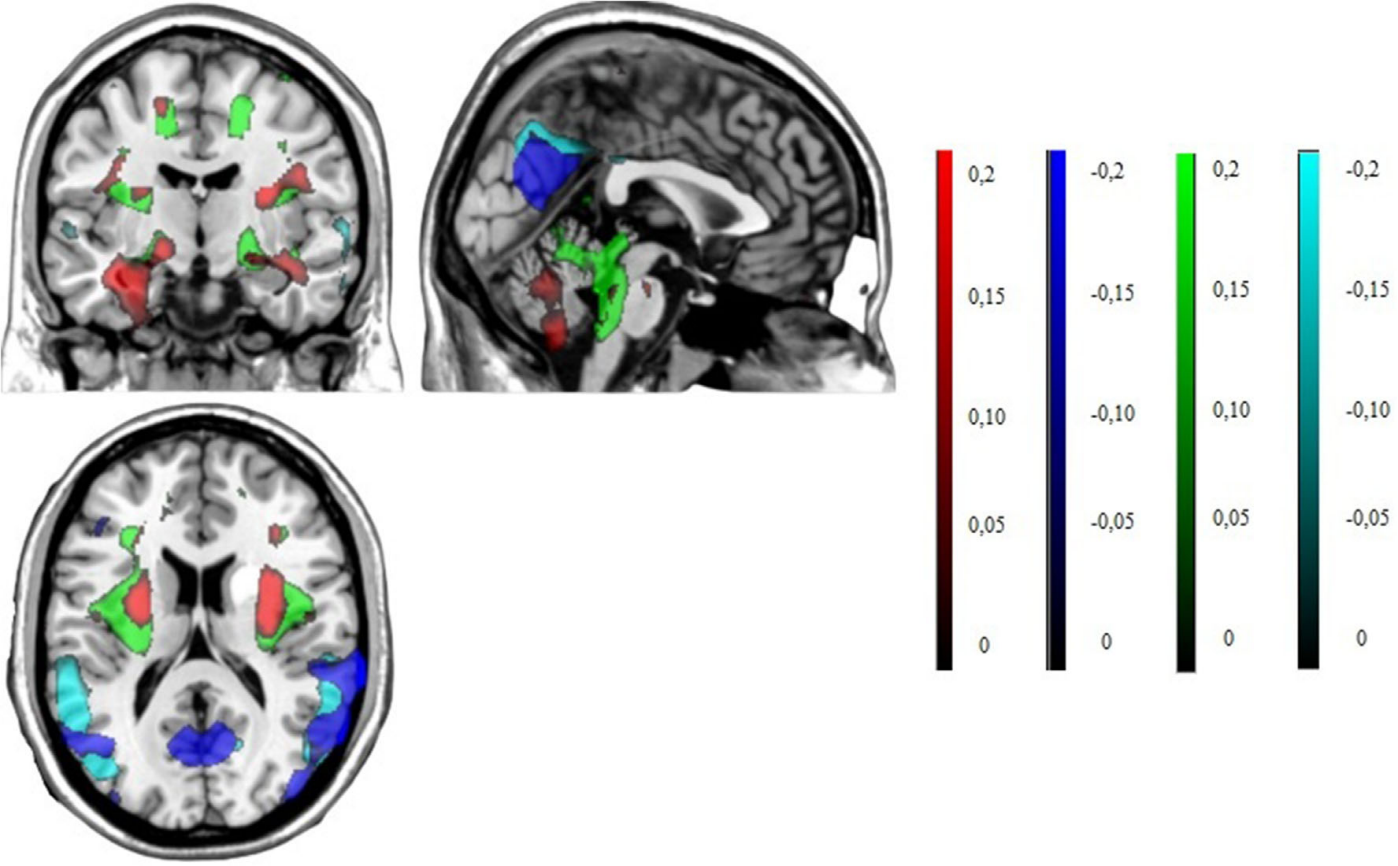
Display of stable voxels of the GE‐iRBDconvRP and RTV‐iRBDconvRP overlaid on a T1‐MRI template in MNI space, determined after bootstrap resampling 95% confidence interval not straddling zero. Red indicates positive voxel weights (relative hypermetabolism), in GE‐iRBDconvRP, whereas green indicates positive voxel weights (relative hypermetabolism) in RTV‐iRBDconvRP. Blue indicates negative voxel weights (relative hypometabolism) in GE‐iRBDconvRP, whereas cyan indicates negative voxel weights (relative hypometabolism) in RTV‐iRBDconvRP. GE, Genoa; iRBDconvRP, phenoconversion‐related pattern of iRBD; MNI, Montreal Neurological Institute; RTV, Rome Tor Vergata. [Color figure can be viewed at wileyonlinelibrary.com]

Areas of overlap among the two independent patterns are shown in Figure 3 and described in Table S4.

**FIG 4:**
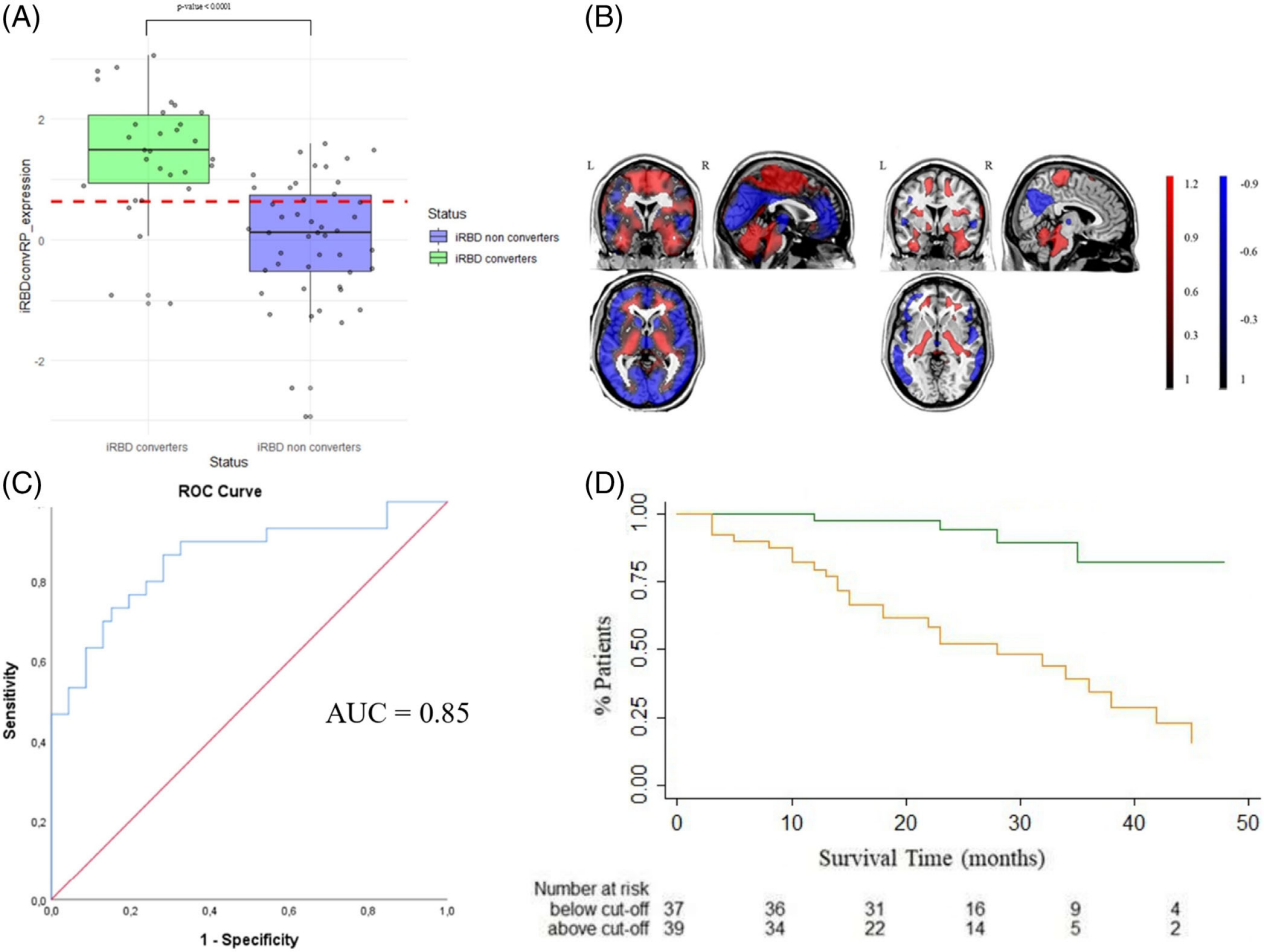
Results of the SSM‐PCA. (**A**) Distribution of subjects’ LOOCV *z* scores of the two groups, with the optimum discrimination threshold (empirical cut point = 0.6414) (green = iRBD converters; blue = iRBD nonconverters). (**B**) Display of the unthresholded iRBDconvRP after SSM‐PCA overlaid on a T1‐MRI template in MNI space (right). Display of stable voxels of the iRBDconvRP overlaid on a T1‐MRI template in MNI space, determined after bootstrap resampling 95% confidence interval not straddling zero (left). Red indicates positive voxel weights (relative hypermetabolism), and blue indicates negative voxel weights (relative hypometabolism). (**C**) Results of ROC analysis of iRBDconvRP (all patients) performed between converter‐ and nonconverter subjects’ LOOCV *z* scores (AUC = 0.85, sensitivity: 87%, specificity: 72%). (**D**) Results of the survival analysis of the iRBDconvRP expression (LOOCV *z* scores). Green line represents iRBDconvRP expression (LOOCV *z* scores) below the empirical optimal cut point. Orange line represents iRBDconvRP expression (LOOCV *z* scores) above the empirical optimal cut point. AUC, area under the curve; iRBDconvRP, phenoconversion‐related pattern of iRBD; LOOCV, leave‐one‐out cross‐validation; MNI, Montreal Neurological Institute; ROC, receiver operating curve. [Color figure can be viewed at wileyonlinelibrary.com]

SSM‐PCA was then applied to the whole cohort. The first two principal components (PC) explained the top 50% of the total variance. A weighted linear combination of PC 1 and 2 (variance explained: 36.42% and 10.59%, respectively) best discriminated between converters and nonconverters in the logistic regression model and was termed the iRBDconvRP. All voxel weights in the iRBDconvRP contributed to the subject scores. Voxels that survived a 2‐tailed CI threshold of 95% (percentile method) after bootstrapping were overlaid on a T1‐MRI template for visualization (Fig. 4B) and included positive voxel weights in the cerebellum, brainstem, anterior cingulate cortex, middle and mesial temporal and postcentral areas, and lentiform nucleus, whereas negative voxel weights were found in the posterior cingulate, precuneus, middle frontal gyrus, and parietal areas (Table S5).

iRBDconvRP subjects’ LOOCV *z* scores were significantly higher in converter than nonconverter patients (*P* < 0.0001; Fig. 4A) as well as in iRBD patients with MCI than iRBD patients without MCI (*P* < 0.01). No significant correlation was found between the iRBDconvRP expression and MDS‐UPDRS‐III score, whereas iRBDconvRP expression showed a significant direct correlation with the presence of MCI (*P* = 0.004).

### iRBDconvRP Phenoconversion Prediction Ability

In the ROC analysis between converter and nonconverter subjects’ LOOCV *z* scores, we found an AUC of 0.85 (sensitivity: 87%, specificity: 72%; Fig. 4C). Kaplan–Meier curves are shown in Figure 4D. The prediction model was statistically significant (*P* < 0.001).

In Cox regression analysis, iRBDconvRP significantly predicted phenoconversion (adjusted HR of 7.42, *P* < 0.001, 95% confidence interval [CI]: 2.5–21.4).

**FIG 5:**
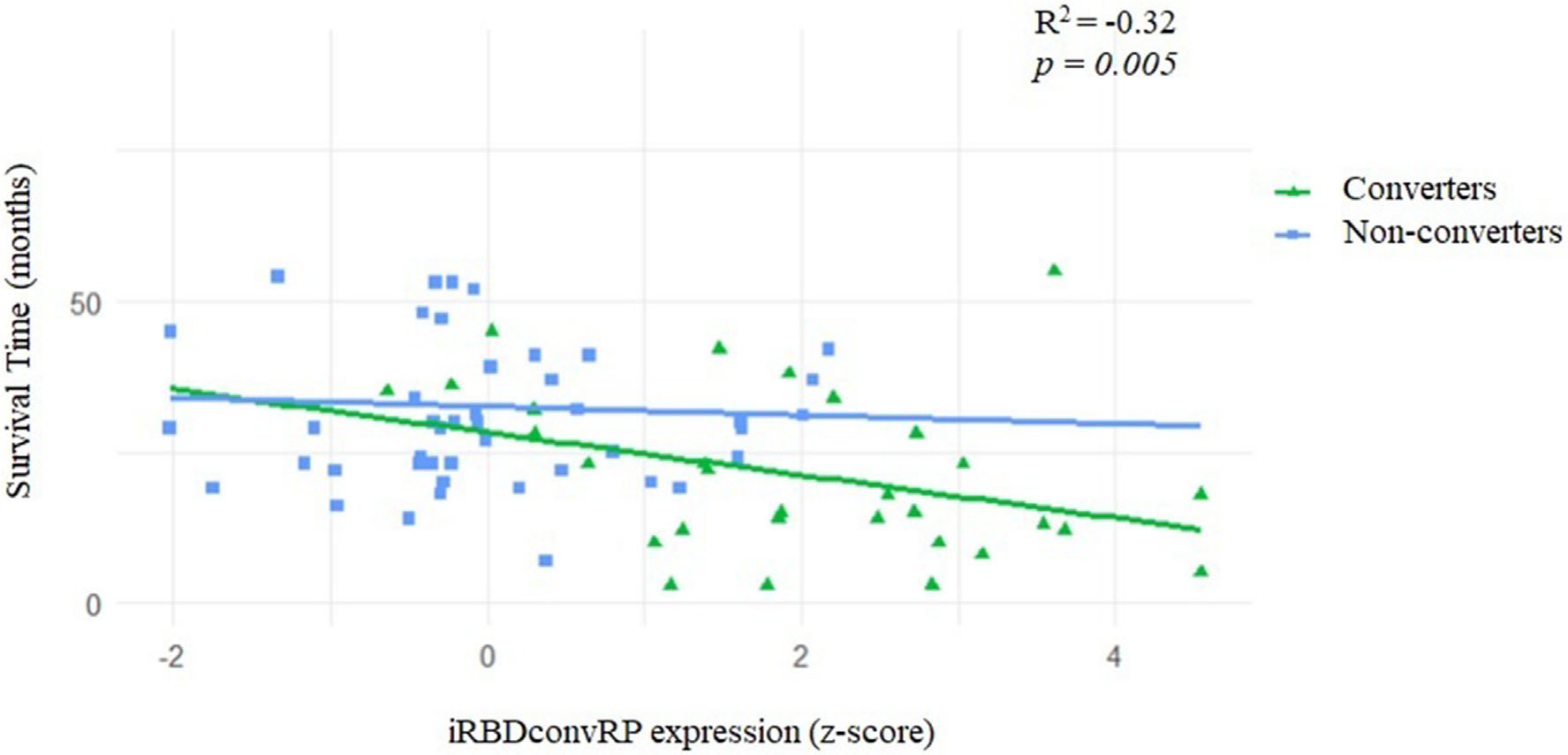
Results of Pearson's correlation between the iRBDconvRP expression (LOOCV *z* score) and survival time (months) in converters (green triangles) and nonconverters (blue squares). iRBDconvRP, phenoconversion‐related pattern of iRBD; LOOCV, leave‐one‐out cross‐validation. [Color figure can be viewed at wileyonlinelibrary.com]

The iRBDconvRP expression showed a significant inverse correlation with survival time (*r* = −0.320, *P* = 0.005; Fig. 5).

The model was significant when MCI and MDS‐UPDRS‐III were included as covariates (HR: 8.88, *P* < 0.001, 95% CI: 2.65–29.79). Besides iRBDconvRP expression, only MDS‐UPDRS‐III score contributed to the prediction with an HR of 1.21 (*P* = 0.028, 95% CI: 1.02–1.44).

It was observed that after the outlier with longer survival time was removed (137 months), the result did not change significantly.

### DenovoPDRBDRP Derivation, Application, and Comparison with the iRBDconvRP

Brain areas involved in the denovoPDRBDRP are presented in Table S6. As expected, these patterns partially overlapped with the iRBDconvRP, with positive voxel weights in the cerebellum and in the anterior cingulate cortex and negative voxel weights in the precuneus and parietal areas, and were significantly and directly correlated (*r*: 0.6, *P* < 0.001).

The denovoPDRBDRP showed a good power in discriminating iRBD converters and nonconverters, with an AUC of 0.85 (sensitivity: 93%, specificity: 67%) and significantly predicted phenoconversion (HR = 3.99, *P* = 0.001, 95% CI: 1.81–8.83). Kaplan–Meier curves are shown in Figure S1.

When combining the Cox regression and the denovoPDRBDRP and the iRBDconvRP, only the latter maintained a statistical significance (HR = 5.72, *P* = 0.003, 95% CI: 1.83–17.85), whereas the former had a HR of 1.81 (*P* = 0.168, 95% CI: 0.78–4.23).

When the time‐dependent ROC curves were compared, the AUC values were higher in the iRBDconvRP than in the denovoPDRBDRP during the first 2 years of follow‐up, whereas the AUC values were higher in the denovoPDRBDRP than in the iRBDconvRP at later time points (Table S7).

## Discussion

In this study, we derived and validated a brain metabolic pattern reflecting the glucose metabolic changes associated with phenoconversion from iRBD to an overt α‐synucleinopathy. Our results reveal the existence of a specific phenoconversion‐related pattern (iRBDconvRP) found by applying the SSM‐PCA to 30 iRBD patients, who phenoconverted to an overt α‐synucleinopathy (14 PD and 16 DLB), and 46 nonconverters. The iRBDconvRP significantly predicted phenoconversion from iRBD to PD or DLB over time, with a high HR.

Initially, because the patients were enrolled by two different centers, two independent iRBDconvRP were derived and cross‐center validated on each other, so as to obtain a more reliable pattern. The resulting patterns were topographically similar and significantly able to predict the conversion in the validation group (ie, GE‐iRBDconvRP on RTV patients and vice versa). It should be noted that the application of RTV‐iRBDconvRP to GE patients showed a lower HR compared with GE‐iRBDconvRP. This finding may be explained by the lower number of patients participating in the derivation of the RTV‐iRBDconvRP. Nevertheless, the RTV‐iRBDconvRP was significantly able to predict conversion in GE patients. This is an intriguing result, considering that no specific harmonization was performed in the [^18^F]FDG‐PET acquisition between the two centers.

The pattern analysis highlights those voxels that either positively or negatively covary together. From a pathophysiological standpoint, we could only speculate that the positive and negative components reflect relatively higher and lower metabolism, respectively. Therefore, the iRBDconvRP includes positive components (relatively higher glucose metabolism) within the cerebellum, brainstem, anterior cingulate cortex, middle and mesial temporal and postcentral areas, and lentiform nucleus, whereas negative components (relatively lower glucose metabolism) were found in the posterior cingulate, precuneus, middle frontal gyrus, and parietal areas.

This pattern partially overlaps with the PDRP described in previous studies, which involves positive voxel weights in the putamen/pallidus, thalamus, pons, and motor cortex and negative components in the premotor cortex, supplementary motor area, and parietal association regions,^14^, ^15^, ^31^ but also shows similarities with the brain glucose metabolism pattern typically found in DLB patients.^35^

It is now recognized that neurodegenerative conditions, such as PD,^2^, ^3^, ^4^ DLB,^35^ and iRBD,^31^ are characterized by disease‐specific patterns, derived from [^18^F]FDG‐PET data.

In particular, it has been shown that the PDRP is more expressed in iRBD patients compared with healthy subjects.^36^ Moreover, the PDRP in iRBD patients is more expressed at follow‐up than at baseline,^30^ suggesting that its expression increases over time, paralleling disease progression from prodromal stages to the overt neurodegenerative disease. Indeed, it has been suggested that the PDRP reflects a more advanced stage of the RBDRP, given the partially overlapped topography.^15^ Interestingly, Kim et al found that RBDRP expression decreases, whereas PDRP expression increases over time, along with disease progression.^16^ Of note, Holtbernd et al^37^ studied the increase in PDRP expression in two groups of iRBD patients (10 and 17 patients, respectively) followed for an average of 5 years. They found an increase in PDRP expression at baseline in both groups, especially in iRBD patients who eventually phenoconverted at follow‐up.

However, iRBD patients are highly heterogeneous,^33^ with about half of them phenoconverting to DLB; thus, a PDRP may not be the best choice to identify subjects at risk of phenoconversion. Here, unlike the aforementioned studies, we wanted to find a pattern reflecting the overall risk of conversion from iRBD to overt alpha‐synucleinopathy in the short‐ to medium term. In fact, iRBD patients who do not convert reflect a heterogeneous group, consisting of late converters and iRBD patients who will truly remain stable (ie, isolated or iRBD). Thus, the pattern derived in this study most likely reflects the risk of short‐ to medium‐term phenoconversion, mean time to conversion being less than 2 years in iRBD converters.

Indeed, a recent multicentric study investigated brain metabolism correlates of DLB core features, showing that the presence of RBD is associated with bilateral parieto‐occipital cortex, precuneus, and ventrolateral–frontal metabolism,^35^ thus in agreement with the iRBDconvRP. To note, the iRBDconvRP overlapped only partially with the previously described RBDRP, which includes positive components within the cerebellum, hippocampus, brainstem, and sensorimotor cortex, as well as negative components within the occipital, temporal, and parietal cortices.^16^, ^31^ Indeed, in the present study, nonconverter iRBD patients rather than healthy controls were used as a reference group to obtain the iRBDconvRP. The reason for this choice was to clean the pattern from the RBDRP components, to obtain a pattern that reflects the stage of the disease, highlighting the metabolic brain areas related to a short‐ to medium phenoconversion, instead of the disease itself.

The iRBDconvRP expression was able to significantly predict phenoconversion in our group of patients, with an adjusted HR of 7.42, thus superior to the already‐known risk factors. Indeed, the clinical risk factors showed HRs of 2 to 5,^2^ whereas presynaptic dopaminergic impairment achieved a HR of 5.71 in a recent multicenter international study.^3^ Interestingly, when used as covariate, the MDS‐UPDRS‐III scores showed a low, but significant power in predicting conversion in iRBD patients. This is not unexpected considering that subtle motor symptoms have been described as predictors of conversion in iRBD^2^ and are likely related to a more advanced stage of the disease. Moreover, the iRBDconvRP showed a higher HR in predicting phenoconversion than the denovoPDRBDRP, as previously described and stated to be a better prediction biomarker than the PDRP.^15^

This result suggests that [^18^F]FDG‐PET may be one among the most accurate biomarkers for the assessment of short to medium term phenoconversion in iRBD patients, thus considered as an inclusion criterion for future disease‐modifying clinical trials. Moreover, the iRBDconvRP expression showed an inverse correlation with survival time (expressed in months and defined as the difference between [^18^F]FDG‐PET acquisition and phenoconversion diagnosis or last outpatient visit), highlighting its possible role as a progression biomarker. However, 90% of iRBD patients will eventually phenoconvert to the overt stage of the disease, when the follow‐up is more than 10 years^38^; thus, the “absence of conversion” that distinguishes nonconverter from converter patients is a condition that is expected to change overtime. Therefore, the iRBDconvRP allows us to identify those patients who will eventually phenoconvert in a short to medium term, because its specificity will be lower for a longer predictive outcome. Nevertheless, in terms of patient selection for neuroprotective trials, the discrimination between long‐ and short‐to‐medium‐term converters could be a useful tool for researchers and clinicians.

In this study, clinical characteristics of iRBD patients are in line with the literature: iRBD converters were older and had more cognitive and motor impairment compared with nonconverters.^2^

Interestingly, RTV patients had more pronounced motor impairments compared to GE patients. Therefore, RTV iRBD patients tended to convert more frequently to PD, whereas GE patients tended to convert more frequently to DLB, although this trend did not reach statistical significance. Despite these differences, the iRBDconvRP well discriminated between converters and nonconverters in both centers, suggesting that the iRBDconvRP represents a stable and reliable pattern, regardless of the phenoconversion diagnosis.

This study has some limitations. First, it has a relatively small sample size. However, this work is the largest [^18^F]FDG‐PET longitudinal study of iRBD patients so far. Second, given the lack of a validation group in the whole cohort, we applied the LOOCV procedure to cross‐validate the results, as done in previous studies.^27^, ^32^ Third, we did not investigate specific differences between converters to DLB and converters to PD because of the limited number of patients and because it was beyond the aim of this study. Fourth, none of the patients developed multiple systemic atrophy, which frequently precedes iRBD, but has a far lower incidence than PD and DLB.^39^ Fifth, because of the lack of an external cohort, longitudinal analyses (ie, Cox regression and time‐dependent ROC) were applied in the same sample used for deriving the iRBDconvRP; therefore, there may be a risk for multicollinearity, and these results should be validated in different cohorts of patients. Finally, the lack of homogeneity exists in [^18^F]FDG‐PET equipment between the two centers. Despite these limitations, finding two similar iRBDconvRP from two different centers, without previous harmonization of the data, may even strengthen our result, suggesting the solidity of the data. Nevertheless, center belonging was used as a nuisance within the analysis. Further longitudinal studies are warranted to confirm these results and eventually to differentiate patterns predicting conversion to different α‐synucleinopathies.

## Author Roles

(1) Research project: A. Conception, B. Organization, C. Execution; (2) Statistical analysis: A. Design, B. Execution, C. Review and critique; (3) Manuscript preparation: A. Writing of the first draft, B. Review and critique.

B.O.: 1A, 1B, 1C, 2A, 2B, 3A

P.M.: 1A, 1B, 1C, 2A, 2B, 3A

C.L.: 1C, 2C, 3B

F.F.: 1C, 2C, 3B

L.G.: 1C, 2C, 3B

A.D.: 1C, 2C, 3B

F.M.: 1C, 2C, 3B

A.G.: 1C, 2C, 3B

D.V.G.: 2C, 3B

S.K.M.: 2C, 3B

K.L.L.: 2C, 3B

F.P.: 1C, 2C, 3B

M.S.: 1C, 2C, 3B

A.C.: 1C, 2C, 3B

R.C.: 1C, 2C, 3B

O.S.: 1C, 2C, 3B

F.I.: 1C, 2C, 3B

N.B.M.: 1C, 2C, 3B

M.P.: 1C, 2C, 3B

M.B.: 1C, 2C, 3B

S.M.: 1C, 2C, 3B

F.N.: 1B, 1C, 2B, 2C, 3A, 3B

D.A.: 1A, 1B, 1C, 2A, 2B, 2C, 3A, 3B

## Supporting information


**APPENDIX S1.** Supporting Information

## Data Availability

The data that support the findings of this study are available from the corresponding author upon reasonable request.

## References

[1] Arnulf I . REM sleep behavior disorder: motor manifestations and pathophysiology. Mov Disord 2012;27(6):677–689. 22447623 10.1002/mds.24957

[2] Postuma RB , Iranzo A , Hu M , et al. Risk and predictors of dementia and parkinsonism in idiopathic REM sleep behaviour disorder: a multicentre study. Brain 2019;142(3):744–759. 30789229 10.1093/brain/awz030 PMC6391615

[3] Arnaldi D , Chincarini A , Hu MT , et al. Dopaminergic imaging and clinical predictors for phenoconversion of REM sleep behaviour disorder. Brain 2021;144(1):278–287. 33348363 10.1093/brain/awaa365 PMC8599912

[4] Zhang H , Iranzo A , Högl B , et al. Risk factors for phenoconversion in REM sleep behavior disorder. Ann Neurol 2022;91(3):404–416. 10.1002/ana.26298 34981563

[5] Antelmi E , Pizza F , Donadio V , et al. Biomarkers for REM sleep behavior disorder in idiopathic and narcoleptic patients. Ann Clin Transl Neurol 2019;6(9):1872–1876. 31386270 10.1002/acn3.50833 PMC6764627

[6] Iranzo A , Fairfoul G , Ayudhaya ACN , et al. Detection of α‐synuclein in CSF by RT‐QuIC in patients with isolated rapid‐eye‐movement sleep behaviour disorder: a longitudinal observational study. Lancet Neurol 2021;20(3):203–212. 33609478 10.1016/S1474-4422(20)30449-X

[7] Arnaldi D , Famà F , Girtler N , et al. Rapid eye movement sleep behavior disorder: a proof‐of‐concept neuroprotection study for prodromal synucleinopathies. Eur J Neurol 2021;28(4):1210–1217. 33275819 10.1111/ene.14664

[8] Iranzo A , Stefani A , Serradell M , et al. Characterization of patients with longstanding idiopathic REM sleep behavior disorder. Neurology 2017;89(3):242–248. 28615430 10.1212/WNL.0000000000004121

[9] Yao C , Fereshtehnejad S‐M , Dawson BK , et al. Longstanding disease‐free survival in idiopathic REM sleep behavior disorder: is neurodegeneration inevitable? Parkinsonism Relat Disord 2018;54:99–102. 29724601 10.1016/j.parkreldis.2018.04.010

[10] Videnovic A , Ju Y‐ES , Arnulf I , et al. Clinical trials in REM sleep behavioural disorder: challenges and opportunities. J Neurol Neurosurg Psychiatry 2020;91(7):740–749. 32404379 10.1136/jnnp-2020-322875 PMC7735522

[11] Pagani M , Nobili F , Morbelli S , et al. Early identification of MCI converting to AD: a FDG PET study. Eur J Nucl Med Mol Imaging 2017;44(12):2042–2052. 28664464 10.1007/s00259-017-3761-x

[12] Meles SK , Pagani M , Arnaldi D , et al. The Alzheimer's disease metabolic brain pattern in mild cognitive impairment. J Cereb Blood Flow Metab 2017;37(12):3643–3648. 28929833 10.1177/0271678X17732508 PMC5718332

[13] Schindlbeck KA , Eidelberg D . Network imaging biomarkers: insights and clinical applications in Parkinson's disease. Lancet Neurol 2018;17(7):629–640. 29914708 10.1016/S1474-4422(18)30169-8

[14] Meles SK , Tang CC , Teune LK , et al. Abnormal metabolic pattern associated with cognitive impairment in Parkinson's disease: a validation study. J Cereb Blood Flow Metab 2015;35(9):1478–1484. 26058693 10.1038/jcbfm.2015.112 PMC4640325

[15] Shin JH , Lee J‐Y , Kim Y‐K , et al. Parkinson disease‐related brain metabolic patterns and neurodegeneration in isolated REM sleep behavior disorder. Neurology 2021;97(4):e378–e388. 34011571 10.1212/WNL.0000000000012228

[16] Kim R , Lee J , Kim YK , et al. Longitudinal changes in isolated rapid eye movement sleep behavior disorder‐related metabolic pattern expression. Mov Disord 2021;36(8):1889–1898. 33788284 10.1002/mds.28592 PMC8451853

[17] Kogan RV , Meles SK , Leenders KL , Reetz K , Oertel WHO . Brain imaging in RBD. Rapid‐Eye‐Movement Sleep Behavior Disorder.Springer International Publishing, Springer; 2019:403–445.

[18] Janzen A , Kogan RV , Meles SK , et al. Rapid eye movement sleep behavior disorder: abnormal cardiac image and progressive abnormal metabolic brain pattern. Mov Disord 2022;37(3):624–629. 34796976 10.1002/mds.28859

[19] Iber C. The AASM Manual for the Scoring of Sleep and Associated Events: Rules, Terminology and Technical Specifications. American Academy of Sleep Medicine 2007.

[20] Gelb DJ , Oliver E , Gilman S . Diagnostic criteria for Parkinson disease. Arch Neurol 1999;56(1):33–39. 9923759 10.1001/archneur.56.1.33

[21] Litvan I , Goldman JG , Tröster AI , et al. Diagnostic criteria for mild cognitive impairment in Parkinson's disease: Movement Disorder Society task force guidelines. Mov Disord 2012;27(3):349–356. 22275317 10.1002/mds.24893 PMC3641655

[22] Postuma RB , Berg D , Stern M , et al. MDS clinical diagnostic criteria for Parkinson's disease. Mov Disord 2015;30(12):1591–1601. 26474316 10.1002/mds.26424

[23] McKeith IG , Boeve BF , Dickson DW , et al. Diagnosis and management of dementia with Lewy bodies: fourth consensus report of the DLB consortium. Neurology 2017;89(1):88–100. 28592453 10.1212/WNL.0000000000004058 PMC5496518

[24] Gilman S , Wenning GK , Pa al L , et al. Second consensus statement on the diagnosis of multiple system atrophy. Neurology 2008;71(9):670–676. 18725592 10.1212/01.wnl.0000324625.00404.15 PMC2676993

[25] Varrone A , Asenbaum S , Vander Borght T , et al. EANM procedure guidelines for PET brain imaging using [18 F] FDG, version 2. Eur J Nucl Med Mol Imaging 2009;36(12):2103. 19838705 10.1007/s00259-009-1264-0

[26] Della Rosa PA , Cerami C , Gallivanone F , et al. A standardized [18 F]‐FDG‐PET template for spatial normalization in statistical parametric mapping of dementia. Neuroinformatics 2014;12(4):575–593. 24952892 10.1007/s12021-014-9235-4

[27] Teune LK , Renken RJ , Mudali D , et al. Validation of parkinsonian disease‐related metabolic brain patterns. Mov Disord 2013;28(4):547–551. 23483593 10.1002/mds.25361

[28] Meles SK , Kok JG , Renken RJ , Leenders KL . From positron to pattern: a conceptual and practical overview of ^18^F‐FDG PET imaging and spatial covariance analysis. In: Dierckx, R.A.J.O., Otte, A., de Vries, E.F.J., van Waarde, A., Leenders, K.L. (eds) PET and SPECT in Neurology. Springer, Cham. 10.1007/978-3-030-53168-3_4

[29] Spetsieris PG , Ko JH , Tang CC , et al. Metabolic resting‐state brain networks in health and disease. Proc Natl Acad Sci 2015;112(8):2563–2568. 25675473 10.1073/pnas.1411011112 PMC4345616

[30] Kogan RV , Janzen A , Meles SK , et al. Four‐year follow‐up of [18F] Fluorodeoxyglucose positron emission tomography–based Parkinson's disease–related pattern expression in 20 patients with isolated rapid eye movement sleep behavior disorder shows prodromal progression. Mov Disord 2021;36(1):230–235. 32909650 10.1002/mds.28260 PMC7891341

[31] Meles SK , Renken RJ , Janzen A , et al. The metabolic pattern of idiopathic REM sleep behavior disorder reflects early‐stage Parkinson disease. J Nucl Med 2018;59(9):1437–1444. 29476004 10.2967/jnumed.117.202242

[32] Meles SK , Kok JG , De Jong BM , et al. The cerebral metabolic topography of spinocerebellar ataxia type 3. NeuroImage Clin 2018;19:90–97. 30035006 10.1016/j.nicl.2018.03.038 PMC6051313

[33] Arnaldi D , Meles SK , Giuliani A , et al. Brain glucose metabolism heterogeneity in idiopathic REM sleep behavior disorder and in Parkinson's disease. J Parkinsons Dis 2019;9(1):229–239. 30741687 10.3233/JPD-181468

[34] Fluss R , Faraggi D , Reiser B . Estimation of the Youden index and its associated cutoff point. Biom J 2005;47(4):458–472. 16161804 10.1002/bimj.200410135

[35] Morbelli S , Chincarini A , Brendel M , et al. Metabolic patterns across core features in dementia with lewy bodies. Ann Neurol 2019;85(5):715–725. 30805951 10.1002/ana.25453

[36] Meles SK , Vadasz D , Renken RJ , et al. FDG PET, dopamine transporter SPECT, and olfaction: combining biomarkers in REM sleep behavior disorder. Mov Disord 2017;32(10):1482–1486. 28734065 10.1002/mds.27094 PMC5655750

[37] Holtbernd F , Gagnon J‐F , Postuma RB , et al. Abnormal metabolic network activity in REM sleep behavior disorder. Neurology 2014;82(7):620–627. 24453082 10.1212/WNL.0000000000000130 PMC3963420

[38] Galbiati A , Verga L , Giora E , Zucconi M , Ferini‐Strambi L . The risk of neurodegeneration in REM sleep behavior disorder: a systematic review and meta‐analysis of longitudinal studies. Sleep Med Rev 2019;43:37–46. 30503716 10.1016/j.smrv.2018.09.008

[39] Vanacore N , Bonifati V , Fabbrini G , et al. Epidemiology of multiple system atrophy. Neurol Sci 2001;22(1):97–99. 11487219 10.1007/s100720170064

