# Supplementary material for: Derivation and Validation of a Phenoconversion‐Related Pattern in Idiopathic Rapid Eye Movement Behavior Disorder
Source: Mov Disord. 2022 Oct 3;38(1):57–67. doi: 10.1002/mds.29236 (PMC10092506; doi:10.1002/mds.29236)
Supplement: Supplementary file 1 — APPENDIX S1. Supporting Information [file MDS-38-57-s001.docx]

Derivation and validation of a phenoconversion-related pattern in idiopathic REM Behaviour Disorder

Pietro Mattioli^1, †^; Beatrice Orso^1,2, †^; Claudio Liguori^3,4,5^; Francesco Famà^1,6^; Laura Giorgetti^6^; Andrea Donniaquio^1^; Federico Massa^1^; Andrea Giberti^1^; David Vallez Garcia^2^; Sanne K. Meles^7^; Klaus L. Leenders^7^; Fabio Placidi^3,4^; Matteo Spanetta^3^; Agostino Chiaravallotti^5^; Riccardo Camedda^5^; Orazio Schillaci^5^; Francesca Izzi^4^; Nicola B. Mercuri^3,9^; Matteo Pardini^1,6^; Matteo Bauckneht^6,10^; Silvia Morbelli^6,10^; Flavio Nobili^1,6^; Dario Arnaldi^1,6^.

1 Department of Neuroscience, Rehabilitation, Ophthalmology, Genetics, Maternal and Child Health (DINOGMI), University of Genoa, Largo Daneo 3, 16132, Genoa, Italy.

2 Department of Radiology and Nuclear Medicine, Amsterdam UMC, Location VuMC, Amsterdam Neuroscience, Amsterdam, the Netherlands.

3 Department of Systems Medicine, University of Rome ‘Tor Vergata”, Rome, Italy

4 Sleep Medicine Center, Neurology Unit, University Hospital “Tor Vergata”, Rome, Italy

5 Department of Biomedicine and Prevention, University of Rome “Tor Vergata”, Rome, Italy

6 IRCCS Ospedale Policlinico S. Martino, Largo Rosanna Benzi 10, 16132, Genoa, Italy.

7 Department of Neurology, University of Groningen, University Medical Center Groningen, Groningen, The Netherlands.

8 Neuroimaging Center, Department of Neuroscience, University of Groningen, Groningen, The Netherlands

9 IRCCS Fondazione Santa Lucia, Rome, Italy

10 Department of Health Science (DISSAL), University of Genoa, Via Antonio Pastore 1, 16132, Genoa Italy.

^†^ These authors contributed equally to this work.

# **Supplementary Materials**

## **Center-specific [^18^F]FDG-PET acquisition.**

As for the GE centre, a SIEMENS Biograph 16 PET/CT hybrid system with a total axial field of view of 15 cm and no interplane gap space was used. To obtain blood glucose level lower than 7.8 mmol/L, patients had to fast for at least six hours before the exam. Once blood glucose level was verified, after a 10 minutes rest in a silent and obscured room, with closed eyes and unplugged ears, 185 – 250 MBq of [^18^F]FDG were injected via a venous cannula. Patients had to stay for 30 minutes in the same room after injection, then they moved to the PET room. Fifteen minutes later PET scans started and lasted ten minutes. A low dose computed tomography transmission scan was performed for attenuation correction. Data were reconstructed using an ordered subset-expectation maximization (OSEM) algorithm, 16 subsets and 6 iterations, with a reconstructed voxel size of 1.33×1.33×2.00 mm.

In RTV, [^18^F]FDG-PET was performed using a PET/CT system Discovery VCT (GE Medical Systems, Tennessee, USA). All the subjects fasted for at least 5 h before intravenous injection of [^18^F]FDG; the serum glucose level was up to 95 mg/mL in all patients. All subjects were injected intravenously with 185–210 MBq of [^18^F]FDG. After the injection all subjects lay down in a semi-darkened, noiseless room, without any artificial stimulation, with their eyes closed. Thirty minutes after [^18^F]FDG injection PET scans started and lasted ten minutes. A low dose computed tomography transmission scan was performed for attenuation correction. Images were reconstructed with an OSEM Algorithm, 20 iterations and 4 subsets, with a reconstructed voxel size of 1.17×1.17×3.27 mm.

## **iRBDconvRP derivation and validation in Genoa Group**

The phenoconversion-related pattern of iRBD (iRBDconvRP) patients was identified by applying an automated algorithm from the University Medical Center Groningen (UCMG), The Netherlands, based on the SSM PCA method of Spetsieris and Eidelberg^1^, implemented in Matlab (version 2020a; MathWorks, Natick, MA) to the [^18^F]FDG-PET data of the Genoa Group patients (GE). The identification method is described in detail elsewhere.^2,3^

For validation, the iRBDconvRP from GE was then used to quantify the [^18^F]FDG-PET scans of the patients from the Rome Tor Vergata (RTV) cohort (14 converters and 19 non-converters). In this procedure, individual scans are projected onto the pattern, resulting in a single score for each scan, this is calculated by multiplying the residual profile for each subject with the GE pattern.^1^

In RTV Group, subject scores in both iRBD non-converter and converter patients were z-transformed to RTV Group iRBD non-converter patients. RTV Group z-scores were then compared between iRBD non-converter and converter patients with a Student’s t test. If significant, the iRBDconvRP was considered valid.

## **iRBDconvRP derivation and validation in Tor Vergata Group**

The same method was applied in RTV group. Once the iRBDconvRP was derived from RTV patients, it was used to quantify the [^18^F]FDG-PET scans of new subjects, in this case GE Group scans (16 converters and 27 non-converters). In this procedure, individual’s scans are projected onto the pattern, resulting in a single score each.^1^

In GE Group, subject scores in iRBD non-converter and converter patients were z-transformed to GE Group iRBD non-converters patients. GE Group z-scores were then compared between iRBD non-converter and converter patients with a Student’s t test. If significant, the iRBDconvRP was considered valid.

**Supplementary Table 1**: Demographic and clinical characteristics of iRBD patients. Values are shown as mean ± standard deviation; median [range].

**Legend:** DLB = Dementia with Lewy Bodies; MDS-UPDRS-III = Movement Disorders Society-sponsored revision of the Unified Parkinson’s Disease Rating Scale, motor section; MMSE = Mini Mental State Examination; PD = Parkinson’s Disease; iRBD = idiopathic REM sleep behaviour disorder; REM = Rapid eyes movements.

**Supplementary Table 2**: Demographic and clinical characteristics of iRBD patients from Genoa (GE) and Rome Tor Vergata (RTV). Values are shown as mean ± standard deviation; median [range].

**Same abbreviations as to Supplementary Table 1.**

**Supplementary Table 3:** Demographic and clinical characteristics of de novo Parkinson’s Disease patients and Healthy Controls. Values are shown as mean ± standard deviation (SD).

|  | De novo PD (n=32) | Healthy Controls (n=44) |
| --- | --- | --- |
| Age (yr) | 73.12 ± 5.86 | 70 ± 8.53 |
| Education (yr) | 10.87 ± 3.85 | 11 ± 3.84 |
| Gender (M:F) | 22:10 | 28:16 |
| MMSE score | 28.09 ± 2.13 | 29 ± 0.78 |
| MDS-UPDRS-III score | 21.12 ± 8.34 | / |
| MCI (Y:N) | 16:16 | / |

**Same abbreviations as to Supplementary Table 1.**

**Supplementary Table 4:** Areas of stable voxels contributing to the GE-iRBDconvRP and RTV-iRBDconvRP, respectively. The described regions are bilateral.
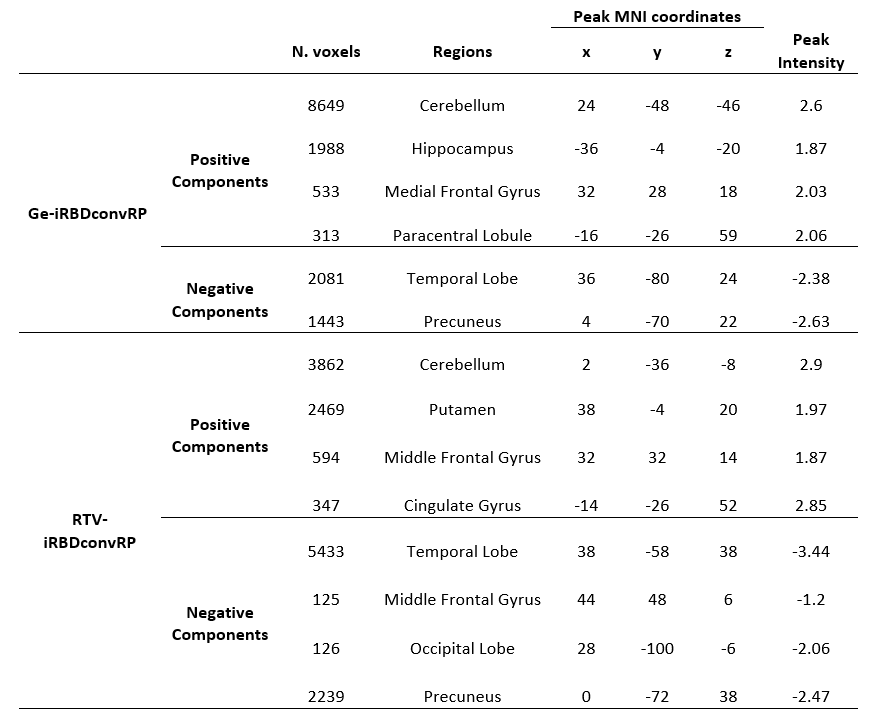


**Supplementary Table 5:** Areas of stable voxels contributing to the iRBDconvRP. The described regions are to be considered as bilateral.


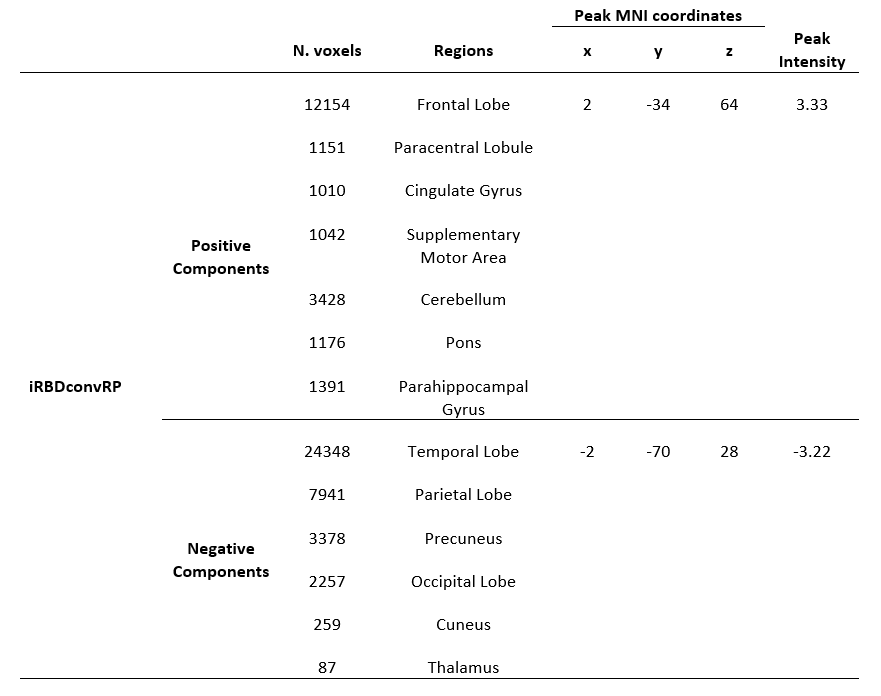


**Supplementary Table 6**. Areas of stable voxels contributing to the denovoPDRBDRP. The described regions are to be considered as bilateral.

|  |  | **N. voxels** | **Regions** | **x** | **y** | **z** | **Peak intensity** |
| --- | --- | --- | --- | --- | --- | --- | --- |
| **denovoPDRBDRP** | **Positive components** | 84 | Left Cerebellum | -4 | -64 | -32 | 1.91 |
|  |  | 63 | Right Cerebellum | 10 | -58 | -34 | 2.13 |
|  |  | 67 | Anterior Cingulate | 10 | 40 | 0 | 2.84 |
|  |  | 97 | Medial Frontal Gyrus | -8 | 40 | -6 | 2.52 |
|  |  | 187 | Putamen | -30 | -6 | 4 | 3.59 |
|  |  | 62 | Superior Temporal Gyrus | 50 | -28 | 4 | 3.86 |
|  | **Negative components** | 378 | Parietal Lobe | 44 | -70 | 40 | -3.64 |
|  |  | 239 | Temporal Lobe | -30 | -78 | 28 | -3.36 |
|  |  | 115 | Precuneus |  |  |  |  |
|  |  | 231 | Occipital Lobe |  |  |  |  |

**Supplementary Table 7**. Results of the time dependant receiver operating curves (ROC) between the iRBDconvRP and the denovoPDRBDRP. Better AUC values for each time-points are reported in **bold**.

| Time-point (months) | iRBDconvRP | denovoPDRBDRP |
| --- | --- | --- |
|  | Area under the Curve (AUC) | |
| 6 | **0.81** | 0.72 |
| 12 | **0.78** | 0.73 |
| 18 | **0.86** | 0.82 |
| 24 | **0.83** | 0.81 |
| 30 | 0.80 | **0.83** |
| 36 | 0.72 | **0.81** |
| 42 | 0.79 | **0.84** |
| 48 | 0.80 | **0.84** |

**Supplementary Figure 1:** Results of the Survival analysis of the denovoPDRBDRP expression on iRBD patients.

Green line = denovoPDRBDRP expression below the empirical optimal cut-point.

Orange line = denovoPDRBDRP expression above the empirical optimal cut-point.


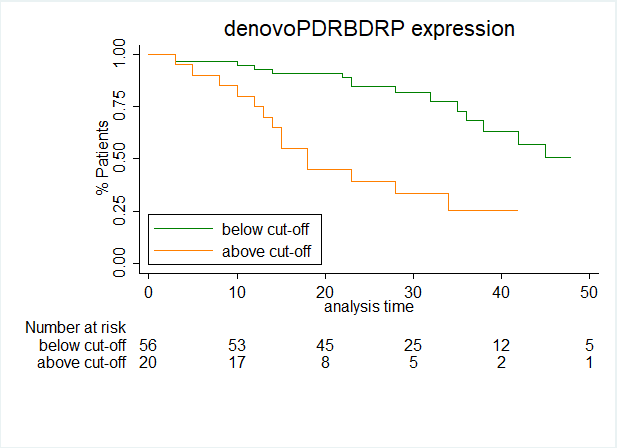


**Supplementary references**

1. Spetsieris PG, Ko JH, Tang CC, et al. Metabolic resting-state brain networks in health and disease. *Proc Natl Acad Sci*. 2015;112(8):2563-2568.

2. Meles SK, Kok JG, De Jong BM, et al. The cerebral metabolic topography of spinocerebellar ataxia type 3. *NeuroImage Clin*. 2018;19:90-97.

3. Meles SK, Renken RJ, Janzen A, et al. The metabolic pattern of idiopathic REM sleep behavior disorder reflects early-stage Parkinson disease. *J Nucl Med*. 2018;59(9):1437-1444.
